# Plateau pika control degrades grasslands while grazing exclusion provides no habitat improvement

**DOI:** 10.1016/j.isci.2026.115159

**Published:** 2026-02-26

**Authors:** Yue Wang, Ning Chai, Wenjin Li, Wenjuan Zhang, Feng Zhang

**Affiliations:** 1 State Key Laboratory of Herbage Improvement and Grassland Agro-ecosystems, College of Ecology, Lanzhou University, 222 Tian Shui South Road, Lanzhou 730000, China; 2 Key Laboratory of Desertification Combating, Gansu Desert Control Research Institute, Lanzhou 730070, China

**Keywords:** environmental science, environmental management, agricultural science

## Abstract

Plateau pika (*Ochotona curzoniae*) control and fencing are primary strategies to combat grassland degradation on the Qinghai-Tibet Plateau. We analyzed 14 years of monitoring data from 1,460 plots across 22 ecoregions to evaluate these interventions. Structural equation modeling revealed that pika suppression exerted a weak indirect negative effect on grassland stability. Neither prolonged pika control nor fencing induced significant overall improvements. While certain grazing regimes initially increased plant richness of both palatable and non-palatable grasses (+18.7%) and non-palatable biomass (+27.4%), these gains diminished after 8–10 years, returning to baseline levels. These findings demonstrate that indiscriminate plateau pika control compromises habitat quality without lasting benefits, and large-scale fencing fails to significantly improve grassland richness or stability. Our results suggest that current reliance on singular interventions is suboptimal, highlighting the need for more integrated and ecologically adaptive management approaches.

## Highlights

• 14-year study shows pika control and fencing fail to enhance alpine meadow stability
• Pika suppression weakens grassland stability, challenging indiscriminate eradication policies
• Fencing shows no long-term improvement in grassland structure or function
• Short-term richness and biomass gains decay to baseline within 8–10 years

## Graphical abstract

**Figure:**
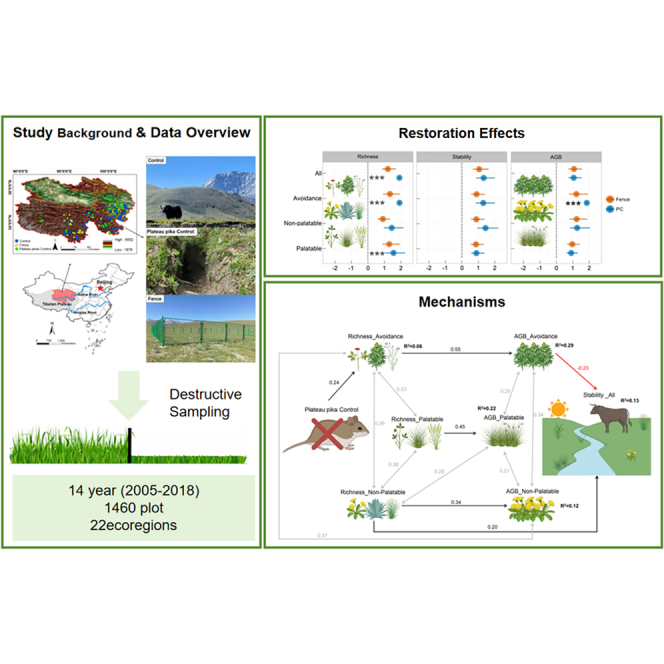

## Introduction

The policy-science nexus constitutes a fundamental tension in environmental governance, wherein the epistemic norms of scientific inquiry conflict with the operational demands of policy-making.^1^^,^^2^ This dichotomy is particularly pronounced in ecosystem management, where complex ecological feedbacks^3^ collide with socioeconomic imperatives for immediate solutions.^1^ China’s extensive pastoral ecosystems (spanning 4 million km^2^) epitomize this challenge, with long-term fencing regimes (>25 years of implementation) and large-scale plateau pika control programs (*Ochotona curzoniae*) dominating grassland restoration efforts since 2000.^4^^,^^5^ Despite significant financial investments (exceeding 12 billion since 2010) and widespread implementation (covering 78% of degraded grasslands), scientific debates persist regarding the ecological rationale and long-term sustainability of these interventions.^6^^,^^7^^,^^8^^,^^9^^,^^10^ Emerging evidence suggests counterintuitive outcomes, including trophic cascade disruptions^3^ and landscape homogenization,^4^ challenging the foundational assumptions of current management paradigms.

Traditional management paradigms have largely characterized plateau pikas as pasture pests that compete with livestock for forage.^3^^,^^11^^,^^12^^,^^13^ This perception has driven extensive control programs. Controlling Brandt’s vole in Mongolia has required annual expenditures ranging from $300,000 to $800,000.^1^^,^^14^ In China, since large-scale rodent control began in the 1960s, the poisons used ranged from zinc phosphide to NH4+–fluoroacetate to the now commonly used anticoagulants (e.g., diphacinone-Na) and botulin toxin C.^15^ As reported by the Chinese Ministry of Agriculture, an annual usage of 38,000 tons of rodenticide was recorded for each year from 2003 to 2005.^3^^,^^16^ However, contemporary ecological research reveals a more nuanced picture. Pikas function as ecosystem engineers through burrowing activities that enhance soil aeration and nutrient cycling.^17^ Their selective foraging may suppress unpalatable plants and facilitate graminoid growth under certain conditions.^18^ This ecological complexity underscores the need to reevaluate pika management strategies.

Grassland fencing persists as a prevalent rehabilitation strategy for degraded pastures, valued for its cost-effectiveness and scalability.^8^^,^^19^ China’s returning grazing land to grassland initiative (2003–present) has fenced 57,600 km^2^ of degraded alpine grasslands in northern Tibet for livestock exclusion.^20^ This was increased in 2011 by a subsidy scheme that compensates herders 90 RMB/ha/year (12 USD/ha/year) for fencing degraded pastures.^5^ However, the ecological benefits of this fencing practice have not been thoroughly evaluated.^3^^,^^5^^,^^8^

Substantial uncertainties persist regarding the spatial scalability and temporal sustainability of current grassland interventions. While small-scale studies abound, comprehensive assessments across multiple ecoregions, and extended time frames remain scarce.^9^^,^^10^ To resolve these questions, we established a longitudinal monitoring network spanning 22 ecoregions (representing 85% of China’s alpine meadow biome) over 14 annual cycles (2005–2018). Through spatially explicit monitoring of 1,460 stratified random plots (mean density: 66.3 plots/region), this study addresses three core questions: (1) how landscape-scale grassland fencing alters vegetation successional trajectories and ecosystem service provisioning; (2) whether sustained plateau pika (*Ochotona curzoniae*) control achieves durable trophic reconfiguration or merely symptom displacement; (3) what temporal thresholds govern management efficacy decay in nonequilibrium grassland systems.

## Results

### The effects of restoration measures on grasslands

**Figure 1:**
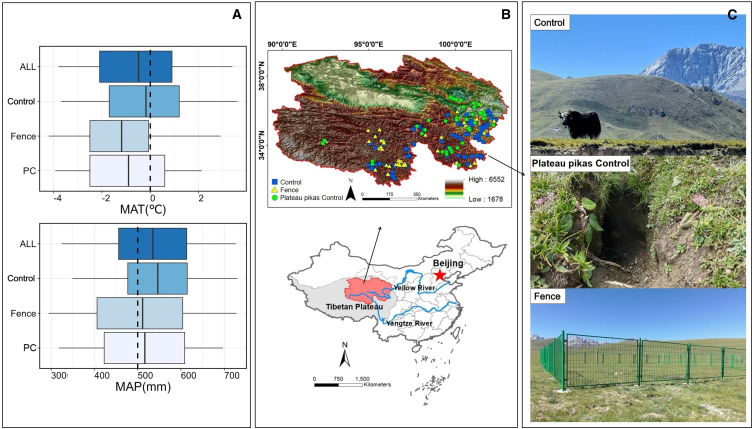
Geographic location and environmental characteristics of the study sites in Qinghai Province (A) Boxplots display the mean annual temperature (MAT) and mean annual precipitation (MAP) across study sites (2005–2018), where boxes represent the median and interquartile range (IQR), and whiskers extend to 1.5 × IQR. (B) Maps show the distribution of the study plots across the Qinghai-Tibet plateau. (C) Representative field photographs illustrate the three management interventions: grazing (control), plateau pika control (PC), and fencing (fence).

**Figure 2:**
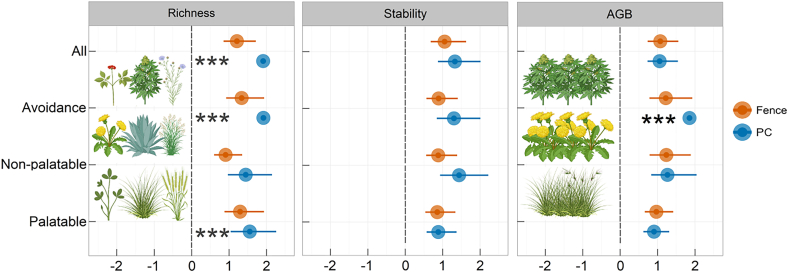
Effects of restoration measures on grassland richness, stability, and aboveground biomass (AGB) Data are represented as mean ± SEM (*n* = 1,460 observations). PC: plateau pika control; fence: grazing exclusion. Asterisks (∗∗∗) indicate statistical significance at *p* < 0.05 using linear mixed-effects models. Images were created using BioRender.com.

The plateau pika control markedly enhanced the total, palatable, and avoidance grassland richness (*p* < 0.05), with effect estimates ranging from 1.56 to 1.92 relative to the control (Figures 1 and 2). On the contrary, there were no differences in grassland richness for the four types (all, avoidance, non-palatable, and palatable) between the fenced and control areas. Similarly, fence did not cause any alterations in terms of grassland stability or aboveground biomass for four types compared to control. In addition, the plateau pika control exhibited a positive effect (est. = 1.85) in aboveground biomass to avoid grassland (Figure 2).

The specific responses of plant communities to management interventions were assessed in two representative plots. In the fenced plot (Banma96), vegetation was dominated by graminoid species such as *Poa annua* and *Elymas nutons* (Table S1), and both species richness and biomass across all palatability classes remained stable annually (Figure S2). In the plot under plateau pika control (Gande85), avoidance grassland accounted for 6.25%–23.53% of the total plant community, with *Oxytropis ochrocephala Bunge* as the dominant avoidance species. This plot also exhibited a significant increasing trend in the richness and biomass of avoidance grasses (Figure S2). Furthermore, the total species abundance increase in the pika-controlled plot (31.25%) exceeded that in the fenced plot (6.25%) (Table S3).

Prolonged fencing maintained overall grassland conditions comparable to the control at a regional scale (see Figure 2; Tables S4 and S5). However, detailed analysis revealed varied ecosystem responses and at 14 of 32 sites, total biomass increased with fencing (*p* < 0.05), while 18 sites saw a significant decrease (*p* < 0.05). Grass richness increased at 13 sites (*p* < 0.05) and decreased at 19 (*p* < 0.05). Stability remained unaffected at all sites relative to controls (*p* > 0.05) (Table S6).The findings indicate that while long-term fencing had minimal impact on grasslands on a larger scale, the effects on grassland ecosystems were heterogeneous on smaller scales.

### Indirect effects of plateau pika control on grassland stability

**Figure 3:**
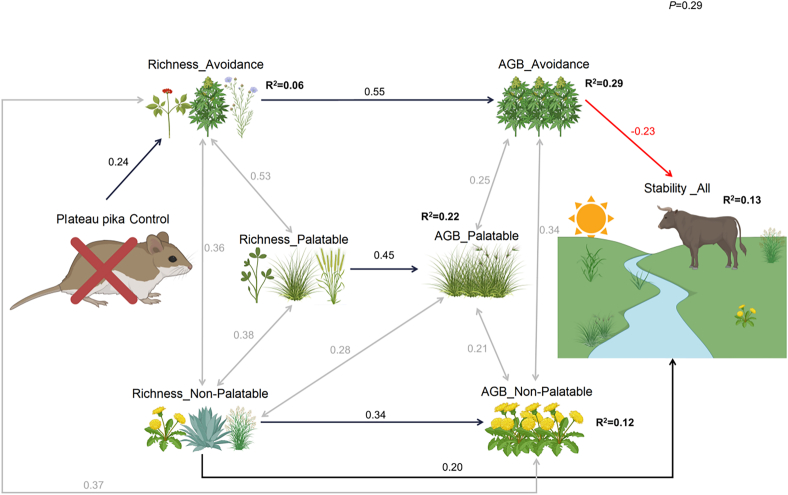
Structural equation modeling of the effectiveness of grassland plateau pika control based on experimental data All arrows represent significant pathways. Coefficients on arrows are standardized regression coefficients (“r” values), mathematical signs indicate positive or negative relationships or a bimodal relationship (±) (*p* = 0.29). Path diagrams were created using BioRender.com.

Increased grassland stability was strongly associated with a decline in avoidance grassland biomass (−0.23) and an increase in non-palatable grassland richness (−0.20) (Figure 3). The control of Plateau pikas had an indirect negative effect on grassland stability, mainly through the effects of resource manipulations on grassland richness avoidance, which in turn affected avoidance grassland biomass, and finally led to a decrease in grassland stability. The models of structural equations demonstrated a statistically significant fit to the data in terms of explaining grassland stability (*p* = 0.29). These models explained 13% of the variation in grassland stability.

### Temporal dynamics of grassland under different measures

**Figure 4:**
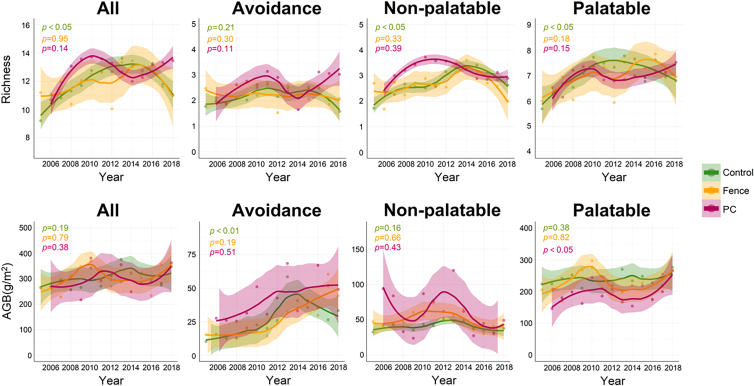
Temporal dynamics of diversity and biomass of different grasslands (all, avoidance, non-palatable, and palatable) under three measures (control (grazing); fence; PC (plateau pika control)) from 2005 to 2018 Shaded areas denote 95% confidence intervals.

Fencing had little impact on grassland richness or stability between 2005 and 2018 (*p* > 0.05). On the contrary, the biomass of palatable grasses increased significantly under the plateau pika control of the plateau (*p* < 0.05). The control of plateau pika also increased grass biomass avoidance in 2011 and 2012 (*p* < 0.05). Overall, the temporal dynamics of the grasslands under fencing and the plateau pika control of the plateau exhibited fluctuating patterns over time (Figure 4; Table S7).

Control treatments led to a significant increase in total grassland, palatable grasses, non-palatable richness, and avoidance grass biomass over the 14-year period (2005–2018), although this was followed by a significant decrease, resulting in an overall increasing trend (Figure 4; Table S7).

## Discussion

### Grazing vs. fencing response

Our findings demonstrate that prolonged fencing maintained grassland conditions comparable to uncontrolled grazing at the regional scale, yet elicited divergent responses at the local level. This scale-dependent response challenges the conventional extrapolation of small-scale fencing studies to broader management contexts. The inconsistent responses across sites, where nearly half showed improved biomass and others declined, demonstrate the limitations of uniform management strategies in heterogeneous landscapes (Table S6).Rather than producing uniformly positive or negative outcomes, the effectiveness of fencing appears to be mediated by local ecological conditions such as vegetation composition, soil properties, and grazing history.

The common practice of generalizing from limited-scale fencing experiments may lead to inaccurate predictions,^21^^,^^22^ as small-scale studies often lack the environmental context necessary for realistic assessment of management interventions.^23^ Instead, we recommend that future assessments incorporate multi-scale sampling designs that adequately account for landscape-level heterogeneity. This approach would provide more reliable guidance for developing targeted conservation strategies that accommodate the ecological variability inherent in grassland ecosystems.

Additionally, it should be noted that our monitoring network was primarily distributed across southeastern Qinghai Province. The northwestern areas consist primarily of uninhabited Gobi desert, which lacks grassland vegetation and is ecologically unsuitable for grazing, precluding data collection in those regions. Given the significant microclimatic diversity of the Qinghai-Tibet Plateau, the general applicability of our conclusions to other regions warrants further investigation. Future research incorporating a broader geographical scope would help validate the universality of these patterns across different eco-climatic zones.

### Grazing vs. plateau pika control response

Plateau pika control, although less frequently studied than fencing or grazing, is widely implemented to protect palatable grasses in various locations. In contrast to grazing, the plateau pika control did not show an impact on palatable grass biomass, but rather facilitated a marked improvement in the richness and aboveground biomass of avoidance grasses (*p* < 0.05). This can be attributed to complementary and selective effects, whereby avoidance grasses capitalize on the ecological niches vacated by palatable grasses that had been suppressed by prolonged grazing under plateau pika control conditions^24^^,^^25^ (Figure S1). However, the increase in grassland avoidance biomass led to a decrease in overall grassland stability (Figure 3). Consequently, the implementation of plateau pika control measures in isolation may be contraindicated, as it could potentially lead to the degradation of wildlife habitats with limited benefits for domestic livestock.

These findings challenge the conventional understanding that plateau pikas contribute to an increase in avoidance grasses and a decrease in palatable grasses^26^^,^^27^^,^^28^^,^^29^ (see Figures 2 and 3; Figure S1; Table S4). The premise of this study was that pikas on the plateau have been presumed to compete with livestock for food and degradation of rangelands. However, the evidence suggests a more nuanced relationship. Plateau pikas exhibit selective foraging behavior and their diet does not necessarily conflict with livestock forage.^30^ Specifically, while primarily consuming grasses and sedges, the plateau pika also feeds extensively on dicotyledonous herbs including legumes. For example, microhistological analysis of stomach contents revealed that *Oxytropis* species (including *O. glabra* and *O. kansuensis*) constituted a substantial dietary component of plateau pikas in *Elymus nutans*-dominated meadows.^31^ Despite the presence of alkaloids and other secondary metabolites in these legumes that typically deter herbivory, plateau pikas appear to counteract these compounds through specialized gut microbiota, enabling their dietary adaptation to the distinct phytochemical composition of high-altitude grasslands.^32^

Their diet exhibits seasonal variation, with higher plant diversity consumed in cold seasons. *Potentilla anserina* contributes notably during this period, demonstrating their adaptive foraging strategy in response to environmental changes. In fact, when overgrazing leads to a proliferation of poorly palatable or avoidance grasses, plateau pikas tend to consume plants that are not preferred by livestock.^3^^,^^33^ Li et al. found that plateau pikas can slow the spread of undesirable plants and promote the growth of graminoids and sedges that are favored by livestock.^33^ Therefore, in well-managed pastures, plateau pikas do not compete with livestock for food. While in heavily degraded rangelands with high plateau pika densities, some competition may occur, but plateau pikas can still provide benefits to livestock production.

It should be noted that the absence of direct field investigation on plateau pika diet constitutes a limitation in this study. Although synthesis of existing literature offers indirect evidence for their dietary plasticity, the lack of primary data restricts mechanistic understanding of interspecific interactions. We thus recommend future employment of DNA metabarcoding for precise diet profiling across regions and seasons, which would strengthen the scientific basis for multi-species management in alpine grasslands.

### Temporal dynamics of grassland under different measures

The findings indicate that the implementation of plateau pika control and fencing did not result in substantial changes in the overall characteristics of the grasslands (Figure 4). However, it should be noted that after the implementation of plateau pika control in 2011 and 2012 (Table S7), there was a significant increase in grassland avoidance biomass. This may be attributed to the capacity of plateau pikas to inhibit the growth of avoidance grasses,^30^ and consequently, the biomass of such plants increased after the plateau pikas population was reduced.

Under grazing conditions (Control), the richness of palatable grasslands, nonpalatable grasslands, and total grasslands, as well as the biomass of avoidance grasses, initially increased but then decreased over time (Figure 4; Table S7). This phenomenon is mainly due to complementary and selection effects. The observed pattern is primarily attributable to the complementary and selective impacts of grazing. With adequate light availability, the ecological niches vacated due to grazing pressure were gradually occupied by restorative vegetation, culminating in augmented grassland richness.^34^^,^^35^^,^^36^ Consequently, due to the selectivity of the livestock foraging, those avoidance grasses by the livestock gradually occupied these vacant ecological niches, resulting in an increase in the biomass of avoidance grasses. Over time, the resilience of the grassland ecosystem caused grassland richness and stability to decline and eventually reached a plateau.

### Limitations of the study

Despite the robust temporal (14 years) and spatial (22 ecoregions) coverage of our monitoring network, several inherent limitations warrant consideration when interpreting these findings. First, while our study captures a broad range of alpine meadow biomes, the geographical focus was primarily centered on southeastern Qinghai Province. This distribution is largely dictated by the fact that the northwestern regions consist primarily of uninhabited Gobi desert and barren land, which lacks the grassland vegetation necessary for grazing and ecological monitoring. Consequently, the successional trajectories identified here may not be directly extrapolatable to these more arid, sparsely vegetated northwestern areas or other distinct high-altitude ecosystems. Second, a notable mechanistic limitation is the absence of primary, site-specific dietary data for plateau pikas from direct field investigations. Although existing literature and ecological indicators provide a basis for our conclusions, the lack of high-resolution diet profiling—such as through DNA metabarcoding—restricts a more granular understanding of the interspecific interactions between pikas and livestock. Finally, the influence of varying local socio-economic dynamics and community compliance on the implementation of these policies was not explicitly quantified, which could introduce additional complexity into the observed ecological outcomes.

## Resource availability

### Lead contact

Further information and requests for resources and materials should be directed to and will be fulfilled by the lead contact, Feng Zhang (zhangfeng@lzu.edu.cn).

### Materials availability

This study did not generate new unique reagents.

### Data and code availability

**Data:** Monthly temperature and precipitation data are based on Peng et al.^29^ Climate datasets (2015–2018) were obtained from the China Meteorological Data Network (http://data.cma.cn). All other ecological monitoring data reported in this study are available from the lead contact upon request.

**Code:** All statistical analyses were performed in R (version 4.3.0). The custom code used for linear mixed-effects modeling and structural equation modeling is available from the lead contact upon request.

**All other items:** Any additional information required to reanalyze the data reported in this paper is available from the lead contact upon request.

## Acknowledgments

This study was supported by Key Laboratory of Desertification Combating (2025-04), the 10.13039/501100001809National Natural Science Foundation of China (grant no 32271761), Gansu Science and Technology Major Project (22ZD6NA007), the “111” Programme (BP0719040), the Fundamental and Interdisciplinary Disciplines Breakthrough Plan of the Ministry of Education of China (JYB2025XDXM910) and this work also supported by the Supercomputing Center of Lanzhou University.

## Author contributions

Conceptualization, data curation, formal analysis, investigation, methodology, writing-original draft, Y.W.; methodology, resources, N.C.; investigation, data curation, validation, funding acquisition, W.L.; resources, supervision, funding acquisition, W.Z.; conceptualization, funding acquisition, project administration, supervision, writing-review & editing, F.Z.

## Declaration of interests

The authors declare no conflict of interest.

## STAR★Methods

### Key resources table

**Table:** 

REAGENT or RESOURCE	SOURCE	IDENTIFIER
**Deposited data**		

Climate data sets (2015-2018)	China Meteorological Data Network	http://data.cma.cn
All ecological monitoring data reported in this study	This paper	N/A

**Software and algorithms**		

R Project for Statistical Computing (v4.3.0)	The R Foundation	https://www.r-project.org/
ArcGIS (v10.7)	Esri	https://www.esri.com/
lme4 R package (v1.1.38)	Bates et al.^37^	https://CRAN.R-project.org/package=lme4
piecewiseSEM R package (v2.3.1)	Lefcheck^38^	https://CRAN.R-project.org/package=piecewiseSEM
dplyr R package (v1.1.4)	Wickham et al.^39^	https://CRAN.R-project.org/package=dplyr
ggplot2 R package	Wickham^40^	https://ggplot2.tidyverse.org
BioRender	BioRender.com	https://biorender.com/

### Experimental model and study participant details

The experimental model in this study involves the plateau pika (*Ochotona curzoniae*), an endemic small mammal of the Qinghai-Tibet Plateau. The influence of sex on the results was not analyzed as management interventions (plateau pika control) were implemented at the population level within specific ecoregions, and the ecological responses were measured at the vegetation community scale. All animal-related field observations were conducted in accordance with institutional permissions and oversight for animal welfare.

### Method details

#### Study site

Situated on the northeast Qinghai-Tibet Plateau (Figure 1), Qinghai Province features a high-altitude environment with an elevational range typically between 3000 and 5000 meters above sea level.^41^^,^^42^ The region exhibits a harsh continental climate, characterized by a subzero mean annual temperature (historically 0°C; -0.5°C during the study period 2005-2018) and an average annual precipitation of 540 mm, with 70% concentrated from June to August (Figure 1). Soils mainly comprise alpine meadow, scrub meadow, and bog types,^43^ supporting characteristic vegetation communities of alpine meadows, shrublands, and steppes dominated by *Carex spp., Kobresia spp., Stipa spp., Achantherum splendens*, and *Potentilla fruticose*.^44^

#### Experimental design

We selected three common management measures: winter and summer grazing pastures (Control), long-term total fencing (Fence), and plateau pika control (Figure 1). In May 2005, 62 experimental areas (each >20 hectares; 500 m × 400 m) were established across multiple counties in Qinghai. Interventions were consistently maintained through multi-year cycles exceeding 3 years (Table S1). These sites characterized the rolling topography and sparse vegetation typical of grazed alpine pastures.

Grazing regimes were aligned with the Chinese agricultural industry code NY/T 635-2015 for carrying capacity. Grassland utilization was standardized across all sites at approximately 1.0 yak/ha (feeding rate 45%–55%), based on guidance from local grassland stations. For grazing exclusion, permanent wire mesh fencing was installed >15 cm above ground to exclude large herbivores while allowing passage for small fauna like microtine rodents.

Thirty pika management sites were selected based on the absence of prior control measures (Table S1). Pika control was applied to peripheral buffer zones exceeding 20 hectares. Within each zone, a central sampling area of <5 hectares was designated for measurements. Population control was executed using botulin C in wheat bait, placed directly into pika burrows.

#### Field survey

In each study area, five randomly placed 10 m × 10 m plots with a minimum spacing of 50 m between the plots were temporally established. All plots were positioned >20 m inward from the habitat boundaries to eliminate the influences of the edge effect. Within each plot, three parallel 1 m × 1 m quadrats were randomly positioned for vegetation sampling (Table S2). The classification of plant species into palatable grasses, non-palatable grasses, and avoidance grasses was based on the dietary preferences of local livestock, particularly yaks. This classification was established through consultation of regional botanical guides combined with interviews with local herders regarding yak foraging behavior.^45^^,^^46^ The metrics of the plant community for each 10 × 10 m plots were calculated as mean values of its three quadrats.

Destructive sampling protocols were implemented by cutting all vegetation at ground level within each quadrat, followed by oven desiccation at 65 °C for 48 hours to achieve constant mass. The dry biomass was then weighed using a precision balance with a resolution of 0.1 g. Systematic annual monitoring occurred during late July to coincide with the phenological peak of vegetation, ensuring standardized comparisons of standing crop biomass across survey cycles.

### Quantification and statistical analysis

#### Grassland index calculation

Species richness was defined as the total number of species identified within each 1 m × 1 m quadrat. Ecosystem stability was illustrated using the Inverse Coefficient of Variation (ICV),^47^ calculated as:

(Equation 1) ICV=μ/σ

where μ is the mean biomass above ground over the experimental years. σ is the standard deviation of the biomass above ground over time.

#### Statistical analysis

Statistical analyses were conducted in R (version 4.3.0). Linear mixed-effects models were implemented using the ‘lmer’ function from the ‘lme4’ package (version 1.1.33). We quantified the effects of climate factors and management measures on richness, stability, and biomass across three palatability categories (avoidance, non-palatable, and palatable). In these models, management measures (control, fencing, and plateau pika control), climate variables (mean annual temperature and annual precipitation), and their interactions were treated as fixed effects, while ‘county’ was treated as a random effect. For all statistical models, the total sample size was n = 1,460 observations, representing the cumulative annual measurements taken across all study plots throughout the experimental period. For all LME results, statistical significance is indicated by asterisks (∗∗∗) representing *p* < 0.05. In all graphical representations, data in summary plots are expressed as mean ± SEM, and temporal dynamics include 95% confidence intervals.

Structural equation modeling (SEM) was performed using the 'psem' function from the ‘piecewiseSEM’ package (version 2.3.0) to analyze the mechanisms underlying changes in grassland stability. Data manipulation was supported by the ‘dplyr’ package (version 1.1.2).^48^ Graphical illustrations were primarily created using BioRender.com.

## Supplemental information

Document S1. Figures S1 and S2 and Tables S1 and S5–S7

Table S2. Plant species collected at the study site

Table S3. Plant Species Palatability to Livestock under Different Management Practices in Representative Plots (PC: Plateau pika Control)

Table S4. Effects of climate and treatment for different grassland compared to the Control (MAT: mean annual temperature; MAP: mean annual precipitation; PC: Plateau pika Control; Control: grazing; ∗∗∗: *p* < 0.05).
