# Supplementary material for: Plateau pika control degrades grasslands while grazing exclusion provides no habitat improvement
Source: iScience. 2026 Feb 26;29(4):115159. doi: 10.1016/j.isci.2026.115159 (PMC12995867; doi:10.1016/j.isci.2026.115159)
Supplement: Document S1. Figures S1 and S2 and Tables S1 and S5–S7 [file mmc1.pdf]

iScience, Volume *29*

Supplemental information

Plateau pika control degrades grasslands while grazing exclusion provides no habitat improvement

Yue Wang, Ning Chai, Wenjin Li, Wenjuan Zhang, and Feng Zhang

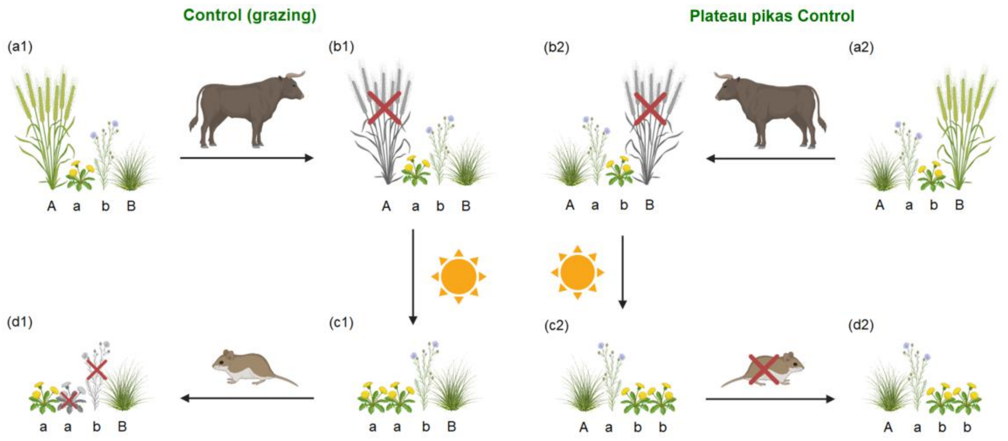

Figure S1. The processes of grassland change under plateau pika control. (a1)(a2) Grasses growing; (b1)(b2) Yaks selectively graze on tall, palatable grasses when grazing; (c1)(c2) According to the ecological niche complementarity effect, under adequate light conditions, community niches vacated by livestock depletion are gradually occupied by restoration vegetation, including palatable grasses and avoidance grasses, but avoidance grasses will gradually take over vacant ecological niches due to the continued grazing pressure on this grassland; (d1) In grasslands without plateau pika control, pika tend to eat avoidance grasses that are not eaten by livestock, and avoidance grasses are reduced; (d2) Plateau pika control in grasslands, maintained after grazing of grasslands (The uppercase letters "ABC" represent palatable grasses and the lowercase letters ab represent avoidance grasses in the figure).

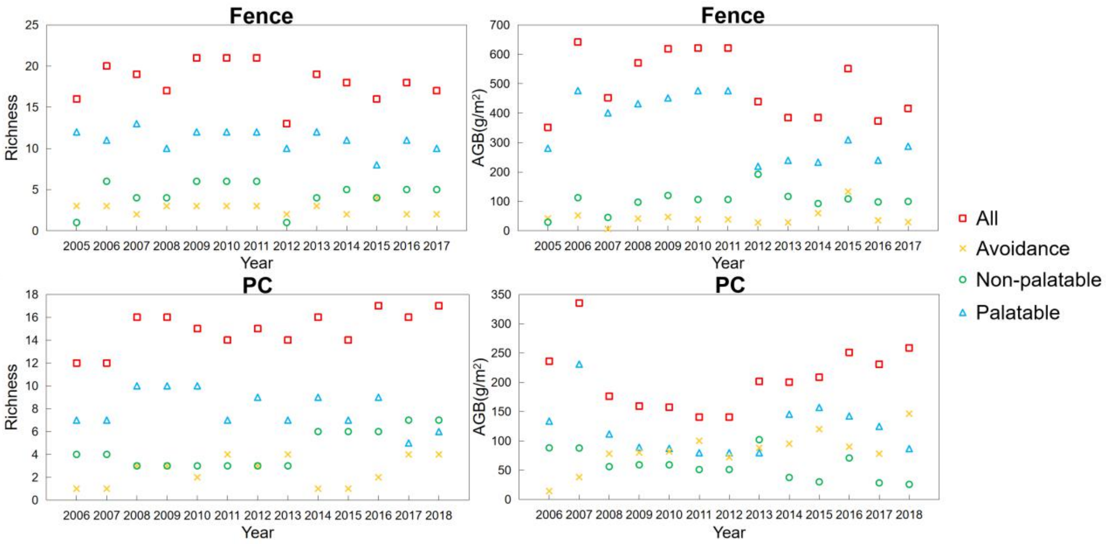

Figure S2. Annual Changes in Species Richness and Biomass of Grassland Plants for Livestock Palatability in Representative Plots (Fence:Banma96, PC:Gande85) under Different Management Practices (PC: Plateau pika Control).

Table S1.Locations of study sites and date of programmes to plateau pika control and fence (PC: Plateau pika Control).

Site number	Site	Longitude(N)	Latitude (E)	country	Time of control	measure
1	Banma93	100.0631111	33.00636111	Banma	May 2005 to October 2017	Fence
2	Banma94	100.0583056	32.99475	Banma	May 2005 to October 2017	Fence
3	Banma95	101.0970833	32.98866667	Banma	May 2005 to October 2017	Fence
4	Banma96	100.8670556	32.82583333	Banma	May 2005 to October 2017	Fence
5	Dari80	98.96916667	33.68694444	Dari	May 2008 to October 2016	Fence
6	Geermu53	92.42788889	34.27602778	Geermu	May 2005 to October 2018	Fence
7	Geermu59	92.42511111	34.21236111	Geermu	May 2006 to October 2016	Fence
8	Geermu61	92.37719444	34.112	Geermu	May 2006 to October 2018	Fence
9	Guide1	101.1236111	36.27416667	Guide	May 2015 to October 2018	Fence
10	Guide5	101.4242	36.355	Guide	May 2015 to October 2016	Fence
11	Maduo68	98.26013889	34.59791667	Maduo	May 2005 to October 2018	Fence
12	Maqin113	99.18522222	34.48177778	Maqin	May 2005 to October 2015	Fence
13	Maqin115	100.0630833	34.52225	Maqin	May 2016 to October 2018	Fence
14	Qumalai45	95.71622222	34.29597222	Qumalai	May 2016 to October 2018	Fence
15	Qumalai46	95.84197	34.24864	Qumalai	May 2016 to October 2018	Fence
16	Qumalai47	94.94905556	34.86	Qumalai	May 2005 to October 2017	Fence
17	Qumalai49	95.34772222	34.55438889	Qumalai	May 2006 to October 2017	Fence
18	Qumalai50	95.36297222	34.54394444	Qumalai	May 2006 to October 2018	Fence
19	Qumalaizeng04	95.36436111	34.52036111	Qumalai	May 2006 to October 2018	Fence
20	Tongdezeng01	100.8465833	34.96016667	Tongde	May 2008 to October 2015	Fence
21	Yushu12	96.75130556	32.90194444	Yushu	May 2005 to October 2018	Fence
22	Yushu14	96.55961111	32.88922222	Yushu	May 2005 to October 2018	Fence
23	Yushu16	97.08527778	32.84388889	Yushu	May 2006 to October 2018	Fence
24	Yushu18	96.69472222	33.12333333	Yushu	May 2006 to October 2018	Fence
25	Yushuzeng01	97.09205556	32.91161111	Yushu	May 2006 to October 2015	Fence
26	Yushuzeng02	96.76861111	33.10138889	Yushu	May 2006 to October 2018	Fence
27	Yushuzeng03	96.01372	32.84478	Yushu	May 2016 to October 2018	Fence
28	Yushuzeng04	96.18614167	32.795	Yushu	May 2015 to October 2018	Fence
29	Zhiduo35	95.76933	33.78747	Zhiduo	May 2015 to October 2018	Fence
30	Zhiduo36	95.58327778	33.81972222	Zhiduo	May 2015 to October 2018	Fence
31	Zhiduo37	95.69416667	33.92141667	Zhiduo	May 2015 to October 2018	Fence
32	Zhiduo39	95.76316667	33.79247222	Zhiduo	May 2015 to October 2018	Fence
33	Banma98	100.2173333	32.84972222	Banma	May 2006 to October 2017	PC
34	Dari73	99.79633333	33.60822222	Dari	May 2007 to October 2018	PC
35	Dari76	99.49466667	33.48866667	Dari	May 2006 to October 2017	PC
36	Dari81	99.74527778	33.725	Dari	May 2006 to October 2016	PC

(Continued)

Site number	Site	Longitude(N)	Latitude (E)	country	Time of control	measure
37	Gande084duizhao	99.66680556	33.84622222	Gande	May 2006 to October 2017	PC
38	Gande83	100.1168889	33.84802778	Gande	May 2006 to October 2015	PC
39	Gande84	99.66680556	33.84622222	Gande	May 2006 to October 2017	PC
40	Gande85	100.3957222	34.13019444	Gande	May 2006 to October 2018	PC
41	Geermu58	92.41683333	34.21888889	Geermu	May 2006 to October 2009	PC
42	Geermu60	92.33011111	34.10772222	Geermu	May 2016 to October 2018	PC
43	Gonghe1	99.75188	36.80255	Gonghe	May 2016 to October 2018	PC
44	Gonghe2	99.70245	36.76705	Gonghe	May 2015 to October 2018	PC
45	Gonghe3	99.97027	36.65208	Gonghe	May 2015 to October 2018	PC
46	Gonghe4	99.90380556	36.68364722	Gonghe	May 2015 to October 2018	PC
47	Gonghe5	100.3203	36.61687	Gonghe	May 2015 to October 2018	PC
48	Gonghe6	100.0340472	36.63711111	Gonghe	May 2015 to October 2017	PC
49	Gonghe8	101.0532	36.43514	Gonghe	May 2015 to October 2018	PC
50	Gonghe9	100.4826944	36.23366667	Gonghe	May 2015 to October 2018	PC
51	Guide2	101.6116667	35.92805556	Guide	May 2015 to October 2018	PC
52	Guide3	101.4575	35.85847222	Guide	May 2015 to October 2018	PC
53	Guide4	101.0758333	36.22055556	Guide	May 2015 to October 2018	PC
54	Jianzha6	101.6892222	35.76925	Jianzha	May 2015 to October 2017	PC
55	Maduo71	98.27016667	34.86236111	Maduo	May 2006 to October 2018	PC
56	Maduo72	98.25352778	34.88825	Maduo	May 2006 to October 2018	PC
57	Maduozeng02	98.05766667	34.68269444	Maduo	May 2009, May 2011 to October 2012, May 2018	PC
58	Tongde125	100.8253611	34.87338889	Tongde	May 2015 to October 2017	PC
59	Tongren6	102.2022333	35.1602	Tongren	May 2015 to October 2017	PC
60	Xinghai152	99.44411111	35.40152778	Xinghai	May 2006 to October 2018	PC
61	Zaduo30	95.20291667	32.91752778	Zaduo	May 2015 to October 2018	PC
62	Zeku141	101.1273611	35.30066667	Zeku	May 2006 to October 2015	PC

Table S5. Tukey HSD test results between different variables for control and fence.

Variables	diff	lwr	upr	*p*
AGB_Avoidance	0.3411645	-10.3467	11.02899	0.94977
AGB_Non-palatable	-2.79081	-16.4385	10.85683	0.686618
AGB_Palatable	-37.79323	-90.1277	14.54125	0.155598
AGB_All	-40.3699	-104.883	24.14352	0.218099
Stability_Avoidance	-0.4092259	-1.76709	0.94864	0.552249
Stability_Non-palatable	-2.706971	-10.5089	5.094917	0.493869
Stability_Palatable	-2.386782	-11.2637	6.490096	0.595857
Stability_All	0.1262085	-0.91173	1.164146	0.810371
Richness_Avoidance	-0.1335315	-0.60268	0.335615	0.574525
Richness_Non-palatable	-0.3966687	-0.9888	0.195464	0.18752
Richness_Palatable	-0.03814705	-0.76975	0.693455	0.918041
Richness_All	-0.5683472	-1.95572	0.819026	0.419364

Notes: diff means average difference between control and fence; lwr means lower bound of the confidence interval; upr means upper bound of the confidence interval.

Table S6.Compared to control, fenced sample plots above or below the variable according to the Tukey HSD.

Variables	Relationship	Plot				
AGB_Avoidance	above	Yushu18	Qumalai47	Banma93	Qumalai49	Yushuzeng01
AGB_Avoidance	above	Guide1	Qumalai45	Dari80	Yushu14	Tongdezeng01
AGB_Avoidance	above	Banma95	Zhiduo37	Yushu12	Yushuzeng02	Banma96
AGB_Avoidance	above	Yushu16	Yushuzeng04	Qumalai50	Yushuzeng03	Maduo68
AGB_Avoidance	below	Geermu61	Geermu59	Geermu53	Maqin113	Guide5
AGB_Avoidance	below	Qumalai46	Maqin115	Zhiduo35	Banma94	Zhiduo39
AGB_Avoidance	below	Zhiduo36	Qumalaizeng04			
AGB_Non-palatable	above	Banma95	Banma96	Dari80	Yushu16	
AGB_Non-palatable	below	Guide1	Qumalai45	Geermu61	Qumalaizeng04	Zhiduo37
AGB_Non-palatable	below	Geermu59	Maqin115	Guide5	Zhiduo35	Geermu53
AGB_Non-palatable	below	Qumalai50	Zhiduo36	Qumalai49	Yushuzeng03	Maduo68
AGB_Non-palatable	below	Banma94	Yushuzeng01	Yushuzeng04	Banma93	Maqin113
AGB_Non-palatable	below	Qumalai46	Zhiduo39	Yushuzeng02		
AGB_Palatable	above	Banma94	Banma93	Yushu14	Banma96	Banma95
AGB_Palatable	above	Yushuzeng02	Yushuzeng04	Yushu16	Yushu18	
AGB_Palatable	below	Geermu59	Geermu53	Maduo68	Zhiduo37	Geermu61
AGB_Palatable	below	Zhiduo35	Maqin115	Zhiduo39	Qumalai46	Guide5
AGB_Palatable	below	Qumalai49	Dari80	Zhiduo36	Qumalai50	Qumalai47
AGB_Palatable	below	Tongdezeng01	Yushuzeng03	Yushu12	Qumalai45	Yushuzeng01
AGB_Palatable	below	Guide1	Maqin113	Qumalaizeng04		
AGB_All	above	Yushuzeng03	Tongdezeng01	Dari80	Banma94	Yushu12
AGB_All	above	Banma93	Yushu14	Yushu18	Banma96	Banma95
AGB_All	above	Yushuzeng04	Yushu16	Yushuzeng01	Yushuzeng02	
AGB_All	below	Geermu59	Geermu53	Geermu61	Zhiduo35	Guide1
AGB_All	below	Maqin115	Qumalai46	Guide5	Zhiduo39	Maqin113
AGB_All	below	Zhiduo36	Qumalai49	Qumalai50	Qumalaizeng04	Qumalai47
AGB_All	below	Zhiduo37	Maduo68	Qumalai45		
Richness_Non-palatable	above	Yushu18	Zhiduo37	Geermu53	Maqin115	Dari80
Richness_Non-palatable	above	Banma95	Banma96	Banma93	Guide5	Banma94
Richness_Non-palatable	above	Yushu14				
Richness_Non-palatable	below	Qumalaizeng04	Qumalai45	Maduo68	Qumalai50	Guide1
Richness_Non-palatable	below	Qumalai47	Qumalai49	Qumalai46	Yushuzeng01	Geermu61
Richness_Non-palatable	below	Yushu16	Zhiduo35	Zhiduo39	Zhiduo36	Yushuzeng02
Richness_Non-palatable	below	Maqin113	Tongdezeng01	Yushu12	Yushuzeng03	Yushuzeng04
Richness_Non-palatable	below	Geermu59				
Richness_All	above	Yushuzeng04	Yushu18	Yushu12	Yushu14	Yushuzeng02
Richness_All	above	Tongdezeng01	Yushu16	Banma95	Banma93	Guide1

(Continued)

Variables	Relationship	Plot				
Richness_All	above	Banma94	Yushuzeng03	Banma96		
Richness_All	below	Qumalai50	Maduo68	Geermu61	Maqin113	Qumalai45
Richness_All	below	Qumalai49	Qumalai47	Qumalaizeng04	Dari80	Guide5
Richness_All	below	Zhiduo39	Qumalai46	Geermu59	Zhiduo37	Zhiduo36
Richness_All	below	Geermu53	Zhiduo35	Yushuzeng01	Maqin115	
Richness_Palatable	above	Geermu53	Maqin115	Yushuzeng04	Yushu14	Qumalaizeng04
Richness_Palatable	above	Tongdezeng01	Yushu18	Zhiduo36	Qumalai46	Yushu16
Richness_Palatable	above	Banma93	Banma94	Yushuzeng03	Banma96	Guide1
Richness_Palatable	above	Yushuzeng02	Banma95			
Richness_Palatable	below	Maqin113	Qumalai50	Dari80	Zhiduo35	Guide5
Richness_Palatable	below	Geermu61	Qumalai47	Geermu59	Yushu12	Qumalai45
Richness_Palatable	below	Qumalai49	Yushuzeng01	Zhiduo39	Maduo68	Zhiduo37
Richness_Avoidance	above	Banma96	Yushuzeng02	Yushuzeng04	Banma93	Tongdezeng01
Richness_Avoidance	above	Yushu12	Guide1	Banma94	Yushu16	Banma95
Richness_Avoidance	below	Guide5	Qumalai46	Geermu61	Maqin113	Qumalai49
Richness_Avoidance	below	Qumalai45	Maqin115	Zhiduo36	Yushu18	Zhiduo35
Richness_Avoidance	below	Maduo68	Qumalai47	Dari80	Qumalai50	Zhiduo37
Richness_Avoidance	below	Geermu59	Yushuzeng01	Geermu53	Yushuzeng03	Yushu14
Richness_Avoidance	below	Qumalaizeng04	Zhiduo39			

Notes: $p < 0.05$: *, $p < 0.01$: ***

Table S7. Temporal dynamics of Richness and biomass of different grassland types (all, avoidance, Non-palatable, palatable) measures (Control (grazing); Fence; PC (Plateau pika Control)) according to the Wilcoxon test.

Measure	Year		All	Avoidance	Non-palatable	Palatable
Control	2006	Richness	*	0.35	*	0.11
Control	2007	Richness	0.19	0.88	0.083	*
Control	2008	Richness	*	0.55	***	0.1
Control	2009	Richness	***	0.56	*	***
Control	2010	Richness	***	*	***	***
Control	2011	Richness	***	*	*	***
Control	2012	Richness	***	0.36	***	***
Control	2013	Richness	***	0.065	***	***
Control	2014	Richness	***	0.44	***	***
Control	2015	Richness	***	*	***	***
Control	2016	Richness	***	*	***	***
Control	2017	Richness	***	0.57	***	***
Control	2018	Richness	*	0.21	*	*
Control	2006	AGB	0.49	0.23	*	0.27
Control	2007	AGB	0.59	0.37	0.092	0.39
Control	2008	AGB	0.81	0.26	0.25	0.79
Control	2009	AGB	0.67	0.33	0.4	0.35
Control	2010	AGB	0.29	***	0.079	0.34
Control	2011	AGB	0.68	***	0.05	0.36
Control	2012	AGB	0.74	***	*	0.34
Control	2013	AGB	0.49	***	*	0.74
Control	2014	AGB	0.82	***	*	0.5
Control	2015	AGB	0.48	***	0.073	0.97
Control	2016	AGB	0.72	***	0.1	0.28
Control	2017	AGB	0.56	***	0.34	0.28
Control	2018	AGB	0.19	***	0.16	0.38
Fence	2006	Richness	0.41	0.56	0.13	0.53
Fence	2007	Richness	0.92	0.34	0.94	0.61
Fence	2008	Richness	0.57	0.27	0.6	0.72
Fence	2009	Richness	0.77	0.46	0.88	0.29
Fence	2010	Richness	0.58	0.57	0.68	0.16
Fence	2011	Richness	0.65	0.71	0.92	0.17
Fence	2012	Richness	0.3	***	0.8	0.99
Fence	2013	Richness	0.26	0.99	0.41	0.16
Fence	2014	Richness	0.21	0.45	0.32	0.17

(Continued)

Measure	Year		All	Avoidance	Non-palatable	Palatable
Fence	2015	Richness	0.3	0.24	0.45	0.14
Fence	2016	Richness	0.14	0.94	0.99	*
Fence	2017	Richness	0.86	0.11	0.98	0.26
Fence	2018	Richness	0.95	0.3	0.33	0.18
Fence	2006	AGB	0.89	0.76	0.87	0.98
Fence	2007	AGB	0.54	0.66	1.1	0.57
Fence	2008	AGB	0.37	0.69	0.78	0.42
Fence	2009	AGB	0.44	0.53	0.46	0.66
Fence	2010	AGB	0.27	0.43	0.46	0.27
Fence	2011	AGB	0.23	0.48	0.11	0.36
Fence	2012	AGB	0.85	0.77	0.99	0.91
Fence	2013	AGB	0.91	0.29	0.63	0.99
Fence	2014	AGB	0.56	*	0.37	0.76
Fence	2015	AGB	0.81	0.099	0.75	0.68
Fence	2016	AGB	0.63	0.09	0.53	0.77
Fence	2017	AGB	0.42	*	0.63	0.47
Fence	2018	AGB	0.79	0.19	0.66	0.82
PC	2007	Richness	0.97	0.94	0.79	0.67
PC	2008	Richness	0.19	0.37	0.15	0.43
PC	2009	Richness	0.079	0.24	0.43	0.22
PC	2010	Richness	0.085	0.27	0.11	0.38
PC	2011	Richness	0.11	0.092	0.19	0.58
PC	2012	Richness	0.14	0.16	0.21	0.62
PC	2013	Richness	0.37	0.53	0.21	0.99
PC	2014	Richness	0.7	0.78	0.28	0.61
PC	2015	Richness	0.17	0.39	0.54	0.5
PC	2016	Richness	0.072	0.069	0.52	0.2
PC	2017	Richness	0.13	0.086	0.25	0.29
PC	2018	Richness	0.14	0.11	0.39	0.15
PC	2007	AGB	0.96	0.92	0.86	0.48
PC	2008	AGB	0.98	0.77	0.48	0.26
PC	2009	AGB	0.72	0.41	0.22	0.43
PC	2010	AGB	0.13	0.096	0.93	0.064
PC	2011	AGB	0.47	*	0.94	0.35
PC	2012	AGB	0.52	*	0.79	0.25
PC	2013	AGB	0.26	0.22	0.33	0.45
PC	2014	AGB	0.73	0.15	0.94	0.5
PC	2015	AGB	0.58	0.43	0.18	0.059
PC	2016	AGB	0.65	0.091	0.7	0.26
PC	2017	AGB	0.54	0.091	0.29	0.11

(Continued)

Measure	Year		All	Avoidance	Non-palatable	Palatable
PC	2018	AGB	0.38	0.51	0.43	*
